# Overexpression of *miR-142-5p* and *miR-155* in Gastric Mucosa-Associated Lymphoid Tissue (MALT) Lymphoma Resistant to *Helicobacter pylori* Eradication

**DOI:** 10.1371/journal.pone.0047396

**Published:** 2012-11-28

**Authors:** Yoshimasa Saito, Hidekazu Suzuki, Hitoshi Tsugawa, Hiroyuki Imaeda, Juntaro Matsuzaki, Kenro Hirata, Naoki Hosoe, Masahiko Nakamura, Makio Mukai, Hidetsugu Saito, Toshifumi Hibi

**Affiliations:** 1 Division of Gastroenterology and Hepatology, Department of Internal Medicine, Shinjuku-ku, Tokyo, Japan; 2 Department of Pathology, Keio University School of Medicine, Shinjuku-ku, Tokyo, Japan; 3 School of Pharmaceutical Sciences, Kitasato University, Minato-ku, Tokyo, Japan; 4 Division of Pharmacotherapeutics, Keio University Faculty of Pharmacy, Minato-ku, Tokyo, Japan; Queen Elizabeth Hospital, Hong Kong

## Abstract

microRNAs (miRNAs) are small non-coding RNAs that can function as endogenous silencers of target genes and play critical roles in human malignancies. To investigate the molecular pathogenesis of gastric mucosa-associated lymphoid tissue (MALT) lymphoma, the miRNA expression profile was analyzed. miRNA microarray analysis with tissue specimens from gastric MALT lymphomas and surrounding non-tumor mucosae revealed that a hematopoietic-specific miRNA *miR-142* and an oncogenic miRNA *miR-155* were overexpressed in MALT lymphoma lesions. The expression levels of *miR-142-5p* and *miR-155* were significantly increased in MALT lymphomas which do not respond to *Helicobacter pylori* (*H. pylori*) eradication. The expression levels of *miR-142-5p* and *miR-155* were associated with the clinical courses of gastric MALT lymphoma cases. Overexpression of *miR-142-5p* and *miR-155* was also observed in *Helicobacter heilmannii*-infected C57BL/6 mice, an animal model of gastric MALT lymphoma. In addition, *miR-142-5p* and *miR-155* suppress the proapoptotic gene *TP53INP1* as their target. The results of this study indicate that overexpression of *miR-142-5p* and *miR-155* plays a critical role in the pathogenesis of gastric MALT lymphoma. These miRNAs might have potential application as therapeutic targets and novel biomarkers for gastric MALT lymphoma.

## Introduction

Extranodal marginal zone B-cell lymphoma of mucosa-associated lymphoid tissue (MALT) is a low-grade lymphoma characterized by histological features such as lymphoepithelial lesions (LELs). The stomach is the most common site of MALT lymphoma, accounting for almost half of all cases. Gastric MALT lymphomas are sometimes associated with chronic inflammation triggered by chronic infection with *Helicobacter pylori* (*H. pylori*), suggesting that the proliferation of MALT lymphoma cells may depend on immune responses to antigens. Indeed, *H. pylori* eradication therapy leads to complete remission in 60–80% of cases of gastric MALT lymphoma and has been used as a first-line treatment [1]–[3]. However, 20–40% of cases do not respond to *H. pylori* eradication therapy, and predictors of the response to antibiotic treatment as well as the appropriate length of the observation period before second-line treatment remain controversial.

The *API2-MALT1* fusion gene, which results from a t(11;18)(q21;q21) translocation, has been identified as the most frequent chromosome translocation in MALT lymphoma cells [4], [5], and Liu *et al.* [6] have reported that it is a potential predictor of resistance to *H. pylori* eradication therapy. Since *API2-MALT1* fusion transcripts lead to inhibition of apoptosis [7], [8], they may confer a survival benefit on MALT lymphoma cells. Despite these reports, the molecular mechanism underlying the initiation and progression of gastric MALT lymphoma is not fully understood.

Many previous studies have focused mainly on aberrant expression of protein-coding genes in the pathogenesis of MALT lymphoma [9]. However, it has recently become apparent that non-coding genes including microRNAs (miRNAs) play important roles as tumor suppressor genes and oncogenes during human carcinogenesis. miRNAs are small (20–25 nucleotides) non-coding RNAs that function as endogenous silencers of target genes. miRNAs are expressed in a tissue-specific manner and play critical roles in cellular proliferation, apoptosis and differentiation [10]. It has been shown that aberrant expression of miRNAs contributes to the development of human malignancies, and that miRNA expression signatures are associated with prognostic factors of human diseases [11]–[15]. Moreover, we have recently proposed that epigenetic regulation of tumor suppressor miRNAs could be a novel therapeutic approach for the treatment of human malignancies [16]–[19].

Although recent studies have shown that *miR-155*, a potential oncogenic miRNA, is highly expressed in diffuse large B cell lymphoma (DLBCL) [20], [21] and overexpression of *miR-155* is correlated with a poor outcome in patients with DLBCL [20], the miRNA expression profiles of low-grade MALT lymphoma have not yet been described. In the present study, therefore, the miRNA expression profiles and potential miRNA target genes of gastric MALT lymphoma were analyzed to clarify the molecular pathogenesis of this malignancy.

## Methods

### Patients and tissue specimens

Twenty patients with primary low-grade gastric MALT lymphomas who were diagnosed and treated at Keio University Hospital (Tokyo, Japan) were enrolled. This study was approved by the ethics committee of Keio University School of Medicine (No. 18-96-3) and was registered with the Clinical Trials Registry (UMIN 000000858). Written informed consent was obtained from all the patients before examination. The clinicopathological and molecular features of the patients are shown in Table 1. The *H. pylori* infection status was identified using the ^13^C-urea breath test. Some cases were confirmed by serological or histological examination in addition to the ^13^C-urea breath test. Tissue specimens from gastric MALT lymphomas and the corresponding non-tumor gastric mucosae were obtained from patients during an endoscopic biopsy and were stored in RNAlater (Ambion, Austin, TX) at −80°C until RNA extraction.

**Table 1:** Clinicopathological and molecular features of gastric MALT lymphoma cases.

			*API2-MALT1*	*H.pylori*	infection	Remission after	Months after
No.	Sex	Age	fusion	before Tx	after Tx	eradication	eradication
1	M	74	+	+	−	NC	74
2	M	74	+	+	−	NC	32
3	M	61	+	−	−	NC	10
4	F	75	+	−	*	NC	*
5	F	59	+	−	*	NC	*
6	F	57	+	−	*	NC	*
7	M	77	−	+	−	NC	45
8	F	68	−	−	−	NC	3
9	F	58	−	+	−	CR	24
10	F	46	−	+	−	CR	10
11	F	66	−	+	−	CR	10
12	F	52	−	+	−	CR	8
13	M	64	−	+	−	CR	6
14	F	71	−	+	−	CR	4
15	F	70	−	+	−	CR	4
16	M	60	−	+	−	CR	4
17	F	56	−	+	−	CR	4
18	M	54	−	+	−	CR	3
19	F	68	−	+	−	CR	2
20	F	40	−	+	−	CR	1

CR, complete remission; NC, no change.

* Eradication therapy not used.

### Fluorescence in situ hybridization (FISH) analysis

To detect the chromosome translocation t(11;18)(q21;q21) and the *API2-MALT1* fusion gene arising from it, FISH analysis using the LSI API2-MALT1 t(11;18)(q21;q21) Dual Color, Dual Fusion Translocation Probe (Vysis/Abbot Laboratories Ltd., Maidenhead, Berkshire, United Kingdom) was performed by Mitsubishi Chemical Medience Corporation (Tokyo, Japan).

### RNA extraction and microarray analysis

Total RNA, including small RNA, was extracted using a mirVana miRNA isolation kit (Ambion). The total RNAs from each of three gastric MALT lymphomas were pooled, as were the total RNAs from each of three matched samples of non-tumor gastric mucosa. The miRNA microarray analysis was performed by LC Sciences (www.lcsciences.com, Houston, TX). RNA from the MALT lymphomas was labeled using Cy5, while the RNA from the non-tumor gastric mucosa was labeled using Cy3. The microarray chip contains probe regions that detect 711 miRNA transcripts listed in Sanger miRBase Release 10.0 (http://www.sanger.ac.uk) and 5 probes for each miRNA. Detected signals greater than background plus 3 times the standard deviation were derived for each color channel; the mean and the co-variance (CV = standard deviation×100/replicate mean) of each probe having a detected signal was calculated. For two color experiments, the ratio of the two sets of detected signals and *p*-values of the *t*-test were calculated. Differentially detected signals were accepted as true when the *p*-values of the ratios were less than 0.01. The average values of their signal intensities are shown in Table 2. All the data were submitted to the ArrayExpress database under the accession number E-MEXP-1898.

**Table 2:** miRNAs differentially expressed between MALT lymphomas and non-tumor gastric mucosae.

No.	microRNA	Non-tumor	MALT	Fold change
		(Cy3 signal)	(Cy5 signal)	
1	*miR-142-5p*	56	1236.3	22.1
2	*miR-142-3p*	25.4	440.5	17.3
3	*miR-223*	574.1	5330.9	9.3
4	*miR-138*	40.9	200.8	4.9
5	*miR-155*	3093.2	13503.9	4.4
6	*miR-572*	1581.7	368.2	0.2
7	*miR-146b-5p*	1029.1	4249.4	4.1
8	*miR-141*	6525.1	1588.8	0.2
9	*miR-146a*	2187.8	8374.4	3.8
10	*miR-378*	3218.7	933.6	0.3

The miRNA microarray analysis was performed by LC Sciences (www.lcsciences.com, Houston, TX). All data were submitted to the ArrayExpress database; the accession number was E-MEXP-1898.

### Quantitative RT-PCR of miRNAs

Levels of miRNA expression were analyzed using quantitative RT-PCR and the TaqMan microRNA assay for *miR-142-5p* and *miR-155* (Applied Biosystems, Foster City, CA), in accordance with the manufacturer's instructions. The expression levels were normalized to that of U6 RNA and expressed as the mean ± standard deviation.

### Infection of C57BL/6 mice with *H. heilmannii*

Ten months prior to the experiment, six-week-old C57BL/6 mice were inoculated with gastric mucosal homogenates from *H. heilmannii*-infected mice, as described previously [22]. Stool DNA was extracted using a QIAamp DNA Stool Mini Kit (Qiagen, Tokyo, Japan), and infection with *H. heilmannii* was confirmed by real-time PCR of stool DNA using specific primers (HeilF, 5′-AAGTCGAACGATGAAGCCTA-3′ and HeilR, 5′-ATTTGGTATTAATCACCATTTC-3′). RNA extraction and histological examination were performed using tissue specimens from the stomachs of *H. heilmannii*-infected mice and control mice. This study was approved by the Keio University Animal Research Committee (No. 08080).

### Western blotting

Protein extracts were separated using SDS/polyacrylamide gel electrophoresis and transferred to a nitrocellulose membrane. The membranes were hybridized with the rabbit anti-human TP53INP1 polyclonal antibody (LifeSpan Biosciences, Seattle, WA). This antibody shows cross-reactivity with mouse TP53INP1. The signal intensities were analyzed using ImageJ software.

### Immunohistochemistry

Formalin-fixed and paraffin-embedded tissues were deparaffinized and rehydrated. For antigen retrieval, the sections were treated for 20 min at 100°C in an autoclave and non-specific reactions were blocked with a blocking reagent (Protein Block Serum-Free, Dako Cytomation, Glostrup, Denmark). The sections were incubated with the rabbit anti-human TP53INP1 polyclonal antibody (diluted 1∶200; LifeSpan Biosciences) overnight at 4°C followed by horseradish peroxidase (HRP)-labeled anti-rabbit IgG (Histofine, Simple stain MAX-PO; Nichirei, Tokyo, Japan) for 30 min at room temperature. Then, the sections were treated with 3, 3′-diaminobenzidine tetrahydrochloride solution. All the sections were counterstained with HE.

### Luciferase assay

Luciferase constructs were made by ligating oligonucleotides containing the wild-type or mutant target site of the *TP53INP1* 3′ untranslated region (UTR) into the *Xba* I site of the pGL3-control vector (Promega, Madison, WI). The AGS human gastric cancer cell line was used in this study. AGS was obtained from the American Type Culture Collection (Rockville, MD). Cells were cultured in RPMI1640 medium supplemented with 10% fetal bovine serum. AGS cells were transfected with 0.4 µg of firefly luciferase reporter vector containing the wild-type or mutant target site and 0.02 µg of the control vector containing *Renilla* luciferase pRL-CMV (Promega) using lipofectamine 2000 (Invitrogen, Carlsbad, CA) in 24-well plates. The *miR-142-5p* and *miR-155* precursor molecules and negative control precursor miRNAs were purchased from Ambion. The molecules were transfected into AGS cells at a final concentration of 100 nM each. The luciferase assays were performed 24 hours after transfection using the Dual Luciferase Reporter Assay System (Promega). The activity of firefly luciferase was normalized to that of *Renilla* luciferase.

### Statistics

Differences in the miRNA levels between the groups were analyzed using paired *t* test and unpaired *t* test. Differences at *p*<0.05 were considered significant.

## Results

### Overexpression of *miR-142-5p* and *miR-155* in gastric MALT lymphoma

To identify miRNAs that play critical roles in the development of gastric MALT lymphoma, we performed miRNA microarray analysis using RNAs obtained from three gastric MALT lymphomas and three matched samples of non-tumor gastric mucosa. The miRNAs that were differentially expressed between non-tumor gastric mucosae and MALT lymphoma lesions are listed in Table 2. The miRNA expression profile revealed that a hematopoietic-specific miRNA, *miR-142* [23], [24], and an oncogenic miRNA, *miR-155* [12], [25], were overexpressed in MALT lymphoma lesions, relative to the levels of expression in the corresponding non-tumor mucosae.

**Figure 1:**
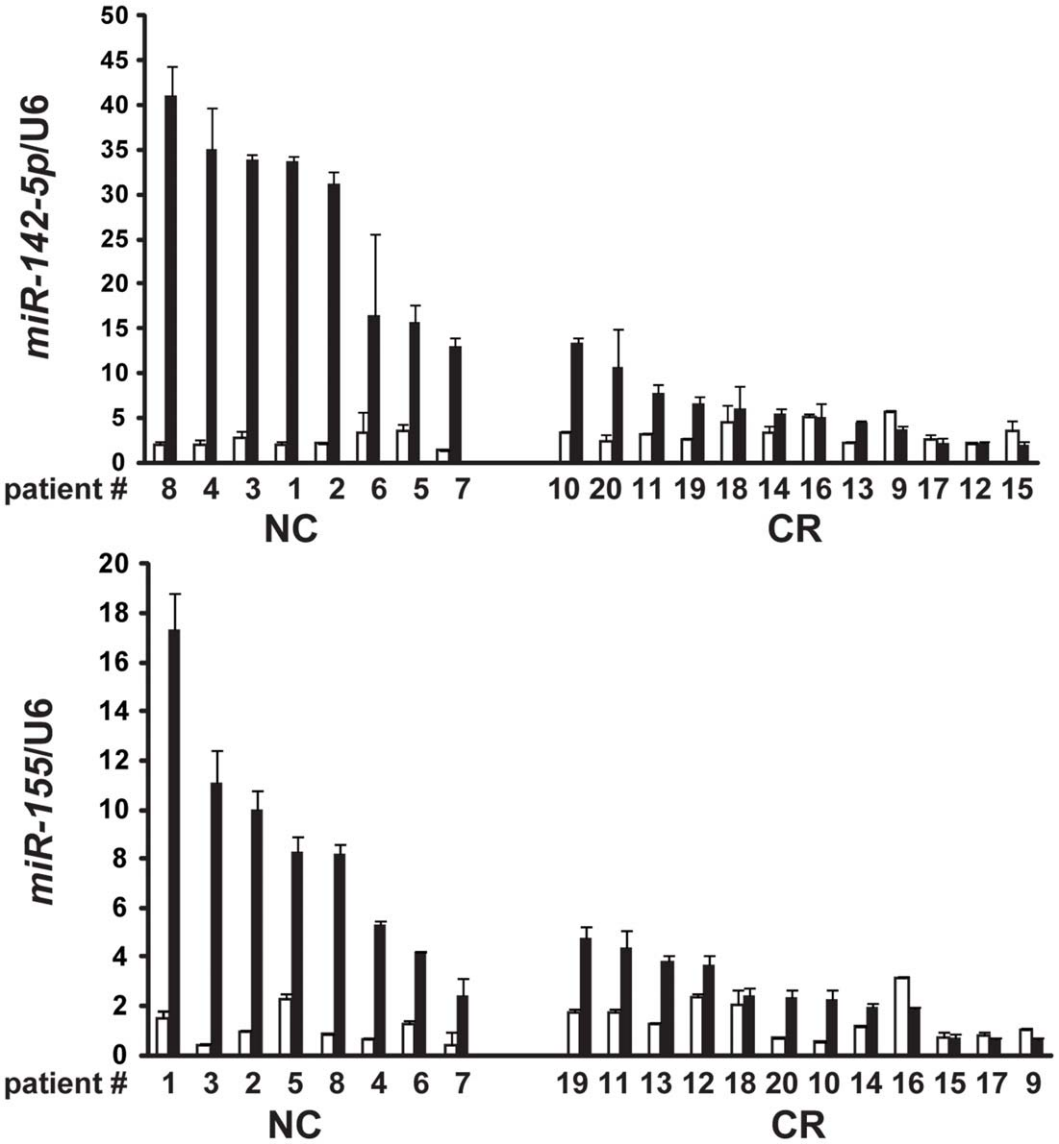
Expression levels of *miR-142-5p* and *miR-155* and responses to *H. pylori* eradication therapy in gastric MALT lymphoma cases. The levels of *miR-142-5p* and *miR-155* expression were evaluated using quantitative RT-PCR and normalized to the expression of U6 RNA. The filled bars and blank bars indicate MALT lymphoma lesions and non-tumor gastric mucosae, respectively. The levels of *miR-142-5p* and *miR-155* expression in gastric MALT lymphoma lesions were significantly increased relative to the corresponding non-tumor gastric mucosae (*p*<0.05 and *p*<0.05, respectively). The levels of *miR-142-5p* and *miR-155* expression in MALT lymphoma lesions which confers resistance to *H. pylori* eradication were significantly higher than those in lesions lacking the *API2-MALT1* fusion gene, which exhibited CR after *H. pylori* eradication (*p*<0.0001 and *p*<0.005, respectively). CR, complete remission; NC, no change.

To validate the microarray data, we performed quantitative RT-PCR for *miR-142-5p* and *miR-155* in 20 cases of gastric MALT lymphoma. The clinicopathological and molecular features of the 20 patients are shown in Table 1. All of the gastric MALT lymphoma cases in the present study were stage I_E_ according to the Ann Arbor staging system [26]. The average age of the patients was 62.5 years (male/female: 7/13). As shown in Figure 1, the expression levels of *miR-142-5p* and *miR-155* in the gastric MALT lymphoma lesions were significantly higher than those in the corresponding non-tumor gastric mucosae (*p*<0.05 and *p*<0.05, respectively).

### *miR-142-5p* and *miR-155* as novel biomarkers of gastric MALT lymphoma

The patients with gastric MALT lymphoma were divided into two groups according to their response to *H. pylori* eradication therapy (Table 1). All the patients who were positive for *H. pylori* underwent eradication therapy and were subsequently confirmed to be *H. pylori*-negative. The use of *H. pylori* eradication therapy for patients with gastric MALT lymphoma who are *H. pylori*-negative is controversial. Nakamura *et al.* have recently reported the long-term clinical outcome of patients with gastric MALT lymphoma after *H. pylori* eradication [27]. In their study, 44 *H. pylori*-negative gastric MALT lymphoma patients underwent *H. pylori* eradication therapy, and 6 (14%) responded to *H. pylori* eradication. Akamatsu *et al.* have also reported that 1 out of 9 (11%) *H. pylori*-negative patients showed complete regression of gastric MALT lymphoma [28]. On the other hand, Fischbach *et al.* have reported that most patients with histological residuals of gastric MALT lymphoma after successful *H. pylori* eradication (treatment failure cases) had a favorable disease course without additional treatment (Fischbach *et al.* Gut 2007; 56: 1685–7). For these patients, a watch and wait strategy with regular endoscopic examinations and biopsies appears to be safe. Our policy of *H. pylori* eradication therapy for gastric MALT lymphoma patients who were *H. pylori*-negative was decided in accordance with the preference of individual patients.

**Figure 2:**
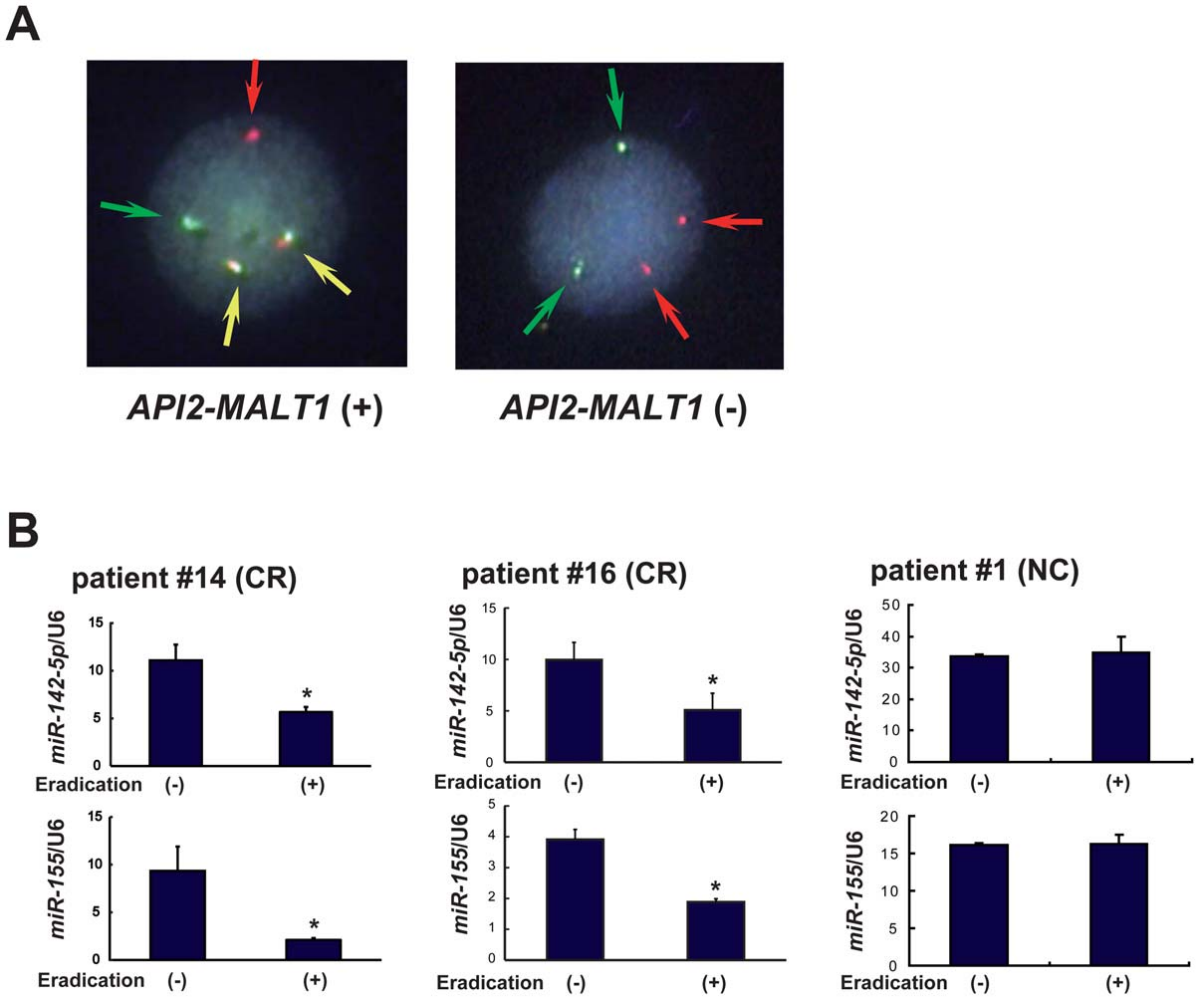
Expression levels of *miR-142-5p* and *miR-155* in gastric MALT lymphoma cases before and after *H. pylori* eradication therapy. (A) Representative cases of the FISH analyses for the detection of the *API2-MALT1* fusion gene are shown. The probes for *API2* and *MALT1* were labeled with green and red signals, respectively. The *API2-MALT1* fusion gene produces a yellow signal. (B) Patients #14 and #16, positive for *H. pylori* and negative for the *API2-MALT1* fusion gene. These patients received *H. pylori* eradication therapy and attained CR. The expression levels of *miR-142-5p* and *miR-155* were significantly lower after *H. pylori* eradication. * *p*<0.05. Patient #1, positive for *H. pylori* and positive for the *API2-MALT1* fusion gene. This patient was resistant to *H. pylori* eradication therapy (NC). The expression levels of *miR-142-5p* and *miR-155* showed no significant differences.

We also analyzed the chromosome translocation t(11;18)(q21;q21) with the *API2-MALT1* fusion gene by FISH analysis. The probes for *API2* were labeled with green signals, and the probes for *MALT1* were labeled with red signals. In positive cases, the *API2-MALT1* fusion genes resulting from the chromosome translocation produced yellow signals (Figure 2A).

As shown in Figure 1, the levels of expression of *miR-142-5p* and *miR-155* in MALT lymphoma lesions that were resistant to *H. pylori* eradication were significantly higher than in cases showing complete remission (CR) after *H. pylori* eradication (*p*<0.0001 and *p*<0.005, respectively). Although the *API2-MALT1* fusion gene has been identified as a potential predictor of resistance to *H. pylori* eradication therapy, patients #7 and #8 with gastric MALT lymphoma lacking the *API2-MALT1* fusion gene were resistant to *H. pylori* eradication. These patients showed increased expression levels of *miR-142-5p* and *miR-155* (Figure 1).

In addition, we investigated the correlation between the levels of *miR-142-5p* and *miR-155* expression and the clinical courses of gastric MALT lymphoma cases. The expression data for patients #14 and #16 with gastric MALT lymphoma who achieved CR after *H. pylori* eradication therapy showed that the levels of *miR-142-5p* and *miR-155* expression were significantly reduced after eradication (Figure 2B, *p*<0.05 and *p*<0.05, respectively). On the other hand, in patient #1 with gastric MALT lymphoma harboring the *API2-MALT1* fusion gene that was resistant to *H. pylori* eradication, there was no significant difference in the expression levels of *miR-142-5p* and *miR-155* (Figure 2B). Although the *H. pylori* infection was eradicated by antibiotic treatment, histological examination showed no regression of the MALT lymphoma lesion. This patient was followed up by observation without treatment, and no marked changes in the endoscopic or histological findings were observed. These findings indicate that the levels of *miR-142-5p* and *miR-155* expression have potential applicability as novel biomarkers of gastric MALT lymphoma.

### Overexpression of *miR-142-5p* and *miR-155* in an animal model of gastric MALT lymphoma

**Figure 3:**
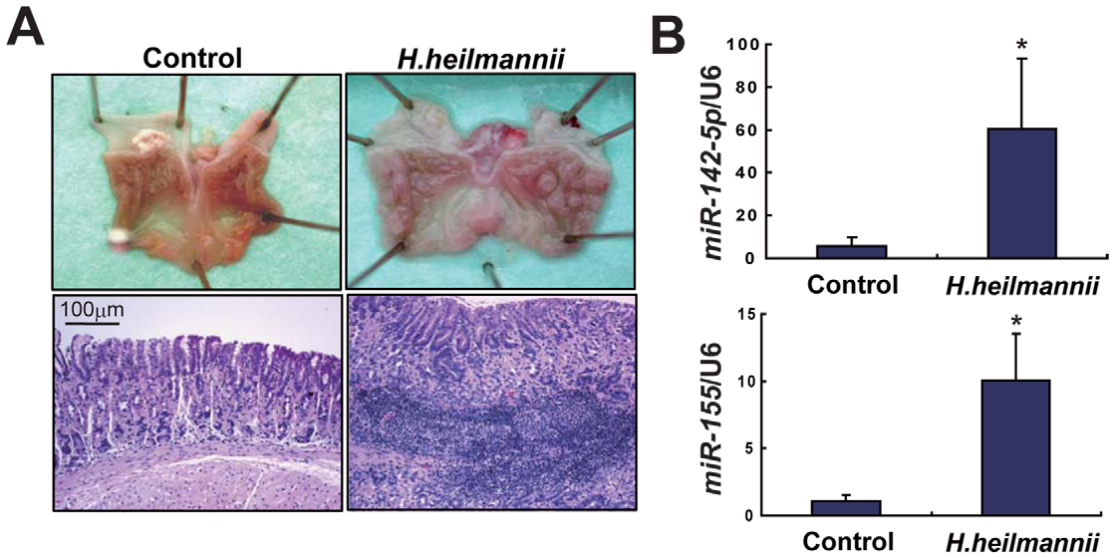
Expression levels of *miR-142-5p* and *miR-155* in an animal model of gastric MALT lymphoma. (A) Macroscopic and light microscopic findings with HE staining of the stomach of control and *H. heilmannii*-infected mice (10 months after infection). Protrusive lesions in the fundic stomach of *H. heilmannii*-infected mice were observed. Light microscopic observations using HE staining revealed the presence of LELs consistent with low-grade MALT lymphoma. (B) Expression levels of *miR-142-5p* and *miR-155* in the stomach of control and *H. heilmannii*-infected mice. The levels of *miR-142-5p* and *miR-155* expression normalized to the level of U6 RNA were significantly increased in *H. heilmannii*-infected C57BL/6 mice. * *p*<0.05.

To further confirm the molecular pathogenesis of gastric MALT lymphoma, we examined the expression levels of *miR-142-5p* and *miR-155* in an animal model of gastric MALT lymphoma. Infection with *H. heilmannii* induces gastric LELs that are consistent with low-grade MALT lymphomas in C57BL/6 mice [22]. Infection with *H. heilmannii* was confirmed using quantitative PCR analysis of stool DNA (data not shown). Ten months after infection with *H. heilmannii*, control (n = 3) and *H. heilmannii*-infected C57BL/6 mice (n = 4) were dissected, and this revealed protruding lesions in the gastric fundus in all of the latter. Light microscopic observations using hematoxylin and eosin (HE) staining revealed the presence of LELs consistent with low-grade MALT lymphomas (Figure 3A). Quantitative RT-PCR analyses showed that the levels of expression of *miR-142-5p* and *miR-155* were significantly higher in *H. heilmannii*-infected C57BL/6 mice (*p*<0.05 and *p*<0.05, respectively), being similar to the results for human gastric MALT lymphomas (Figure 3B).

### *miR-142-5p* and *miR-155* suppress the proapoptotic gene *TP53INP1* as their target

Identification of miRNA target genes is essential for determining miRNA function. Recent studies have indicated that a single miRNA may regulate more than 200 target genes. A database for predicting target genes, TargetScan (http://www.targetscan.org), revealed that both *miR-142-5p* and *miR-155* are able to bind to the 3′ UTR of the mRNA of the proapoptotic gene *TP53INP1* (Tumor Protein P53 Inducible Nuclear Protein 1). Moreover, *miR-155* has been shown to repress *TP53INP1*, which inhibits the development of pancreatic tumors [29]. Therefore, we focused on *TP53INP1* as a common target of *miR-142-5p* and *miR-155*.

**Figure 4:**
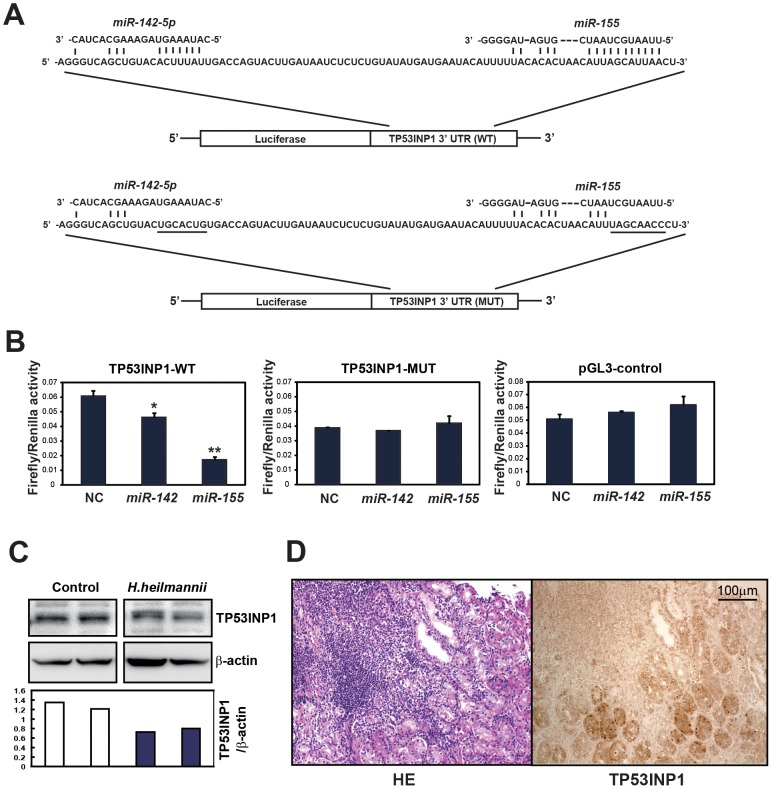
*miR-142-5p* and *miR-155* suppress *TP53INP1* as their target. (A) Luciferase reporter constructs of wild-type (WT) and mutant (MUT) target sites in the 3′ UTR of *TP53INP1* mRNA. The base pairings between *miR-142-5p* and *miR-155* and their putative target sites in the 3′ UTR of *TP53INP1* mRNA are shown. Underlining indicates mutant sequences. (B) Firefly luciferase activity was normalized to the *Renilla* luciferase activity of *TP53INP1* wild-type (WT), mutant (MUT), and pGL3 control (empty vector) in AGS cells transfected with negative control (NC), *miR-142-5p*, and *miR-155*. The luciferase activities of the AGS cells transfected with the *TP53INP1*-WT construct were significantly lower after transfection of *miR-142-5p* and *miR-155*, whereas those transfected with the *TP53INP1*-MUT construct or the pGL3 control vector (empty vector) showed no significant differences. * *p*<0.05 and ** *p*<0.005, compared with negative control. (C) Western blot analysis of TP53INP1 in control and *H. heilmannii*-infected mice. β-actin was used as a loading control. The expression of TP53INP1 was suppressed in *H. heilmannii*-infected mice, compared with the expression level in control mice. (D) Immunohistochemistry for TP53INP1 in human gastric MALT lymphoma. Sections were counterstained with HE. TP53INP1 staining was markedly reduced in the MALT lymphoma lesions.

To confirm the target specificity of *miR-142-5p* and *miR-155* for *TP53INP1*, we performed a luciferase reporter assay using a vector containing the putative *TP53INP1* 3′ UTR target sites downstream of the luciferase reporter gene, which was transfected into AGS cells. The base pairing between *miR-142-5p* and *miR-155* and the wild-type (WT) or mutant (MUT) target sites in the 3′ UTR of *TP53INP1* mRNA is shown in Figure 4A. The luciferase activities of the AGS cells transfected with the *TP53INP1*-WT construct were significantly lower after transfection with *miR-142-5p* and *miR-155* (*p*<0.05 and *p*<0.005, respectively), whereas those of cells transfected with the *TP53INP1*-MUT construct and the pGL3 control vector (empty vector) showed no significant differences (Figure 4B). It has been shown that conserved perfect 6- to 8-bp matches between the 5′ end of the mature miRNA and the 3′ UTR of the predicted target mRNA (called ‘seed’ matches) are the most important factor determining miRNA targets [30]. As shown in Fig. 4A, ‘seed’ matches between the 5′ end of the miRNAs and the 3′ UTR of *TP53INP1* were stronger in *miR-155* than in *miR-142*, suggesting that *miR-155* may have a more profound effect on suppression of TP53INP1.

We next examined the expression levels of TP53INP1 by Western blot analysis in *H. heilmannii*-infected mice and immunohistochemistry in human gastric MALT lymphoma-like lesions. The expression of TP53INP1 was suppressed in *H. heilmannii*-infected mice, relative to control mice (Figure 4C). We examined the expression levels of TP53INP1 in human gastric tissues by immunohistochemistry. The gastric tissue shown in Fig. 4D contains both MALT lymphoma and non-MALT lymphoma tissue. The infiltrating lymphocytic lesion shown by HE staining is consistent with MALT lymphoma. TP53INP1 staining is negative or faint in the MALT lymphoma lesion, whereas normal gastric glands around the MALT lymphoma show sufficient staining for TP53INP1. Thus TP53INP1 staining was markedly reduced in MALT lymphoma lesions (Figure 4D). These findings suggest that *TP53INP1* is a common target of *miR-142-5p* and *miR-155* and is suppressed by overexpression of both *miR-142-5p* and *miR-155* in gastric MALT lymphoma lesions.

## Discussion

A recent study has demonstrated that miRNA expression profiles can be used to classify the developmental lineages and differentiation stages of tumors, and are more accurate for tumor classification than conventional mRNA profiles [31]. Furthermore, miRNA expression signatures are associated with prognostic factors and disease progression in chronic lymphocytic leukemia [11] and lung cancer [32]. Thus miRNA expression is clinically promising as both a diagnostic tool and a prognostic marker for human malignancies. The results of microarray analysis revealed a unique miRNA expression profile in gastric MALT lymphoma. Recent studies have demonstrated that the expression level of *miR-155* is significantly elevated in DLBCL, which is considered to represent high-grade transformation from MALT lymphoma [20], [33]. *miR-142*, *miR-155* and *miR-223* have been reported to be hematopoiesis-specific miRNAs [24]. These findings are consistent with our results, and in the present study we focused on *miR-142* and *miR-155*, because *miR-142* is the most up-regulated miRNA and *miR-155* plays a critical role in the pathogenesis of B-cell lymphoma. The levels of *miR-142-5p* and *miR-155* expression were associated with the clinical course of gastric MALT lymphoma, including the response to *H. pylori* eradication. The *API2-MALT1* fusion gene has been identified as a potential predictor of resistance to *H. pylori* eradication therapy. In the present study, two cases of gastric MALT lymphoma resistant to *H. pylori* eradication lacked the *API2-MALT1* fusion gene (patients #7, #8). These cases showed increased expression of *miR-142-5p* and *miR-155*. Therefore, these miRNAs may be more useful markers than the *API2-MALT1* fusion gene in patients with gastric MALT lymphoma.

*TP53INP1* is a proapoptotic stress-induced p53 target gene. p53 activates *TP53INP1* transcription, and overexpression of *TP53INP1* induces cell cycle arrest and apoptosis [34]. Gironella *et al.* [29] have shown that oncogenic *miR-155* is overexpressed in pancreatic ductal adenocarcinoma and suppresses its target, *TP53INP1*, resulting in cancer progression. In agreement with these findings, our results showed that *TP53INP1* was suppressed by both *miR-142-5p* and *miR-155*, possibly leading to inhibition of apoptosis and acceleration of MALT lymphoma cell proliferation. These findings suggest that overexpression of *miR-142-5p* and *miR-155* concomitant with suppression of *TP53INP1* reflect the increased proliferation of MALT lymphoma cells. The distinct connection between aberrant expression of *miR-142-5p* and *miR-155* and the progression of MALT lymphoma suggests that miRNAs could be potential therapeutic targets. A recent study has shown that chemically engineered oligonucleotides, termed ‘antagomirs,’ can work as specific inhibitors of endogenous miRNAs in mice [35], and might be potentially applicable to silence *miR-142-5p* and *miR-155* for the treatment of gastric MALT lymphomas that are resistant to *H. pylori* eradication therapy.

## Conclusions

Overexpression of *miR-142-5p* and *miR-155* is presumed to play a critical role in the initiation and progression of gastric MALT lymphoma, suggesting that these miRNAs may be potentially useful as therapeutic targets and novel biomarkers for gastric MALT lymphomas. Inhibition of *miR-142-5p* and *miR-155* might be a novel approach for the prevention and treatment of gastric MALT lymphoma. Further studies involving more patients are warranted to explore the clinical use of *miR-142-5p* and *miR-155* for diagnosis and treatment of gastric MALT lymphoma.
