# Correction: Overexpression of *miR-142-5p* and *miR-155* in Gastric Mucosa-Associated Lymphoid Tissue (MALT) Lymphoma Resistant to *Helicobacter pylori* Eradication

**DOI:** 10.1371/annotation/53d1898f-d0ae-4e5c-a585-c084a5c881bf

**Published:** 2013-07-31

**Authors:** Yoshimasa Saito, Hidekazu Suzuki, Hitoshi Tsugawa, Hiroyuki Imaeda, Juntaro Matsuzaki, Kenro Hirata, Naoki Hosoe, Masahiko Nakamura, Makio Mukai, Hidetsugu Saito, Toshifumi Hibi

There was an error in affiliation 1 for authors Yoshimasa Saito, Hidekazu Suzuki, Hitoshi Sugawa, Hiroyuki Imaeda, Juntaro Matsuzaki, Kenro Hirata, Naoki Hosoe, Hidetsugu Saito and Toshifumi Hibi. The correct affiliation is: 1. Division of Gastroenterology and Hepatology, Department of Internal Medicine, Keio University School of Medicine, Shinjuku-ku, Tokyo, Japan. 

